# Natural-Origin Edible Gels as Delivery Systems for Green Tea Extract: Formulation, Physicochemical, and Biopharmaceutic Profile Assessment

**DOI:** 10.3390/molecules30132789

**Published:** 2025-06-28

**Authors:** Andreja Poceviciute, Agne Mazurkeviciute, Lina Raudone

**Affiliations:** 1 Laboratory of Biopharmaceutical Research, Institute of Pharmaceutical Technologies, Lithuanian University of Health Sciences, Sukileliu av. 13, LT-50162 Kaunas, Lithuania; andreja.poceviciute@stud.lsmu.lt (A.P.); agne.mazurkeviciute@lsmu.lt (A.M.); 2 Department of Pharmacognosy, Lithuanian University of Health Sciences, Sukileliu av. 13, LT-50162 Kaunas, Lithuania

**Keywords:** *Camellia sinensis*, catechins, caffeine, natural gelling agents, flaxseed, oat

## Abstract

Natural-origin edible gels are gaining attention as innovative carriers for bioactive compounds, offering consumer-friendly formats and potential to enhance stability and bioavailability. This study aimed to develop and characterize edible gels incorporating *Camellia sinensis* (L.) Kuntze extract using different plant-based gelling agents, including whole flaxseeds, ground flaxseeds, medium-size oatmeal, and coarse oatmeal. The physical properties of the gels were evaluated by rheological (flow curve) and pH studies. The phytochemical composition of the green tea extract and gels with this extract and the main phenolic compounds, including catechins, gallic acid, and caffeine, were evaluated by high-performance liquid chromatography. The biopharmaceutical properties of the prepared gels were evaluated by dissolution testing. Rheological analysis revealed that oat-based gels exhibited higher viscosity (up to 24.33 Pa·s) compared to flaxseed-based gels. Despite differences in consistency, no statistically significant differences were found in total phenolic release among gel formulations (*p* > 0.05), except for epigallocatechin, which showed significantly higher release from coarse oatmeal gels (*p* > 0.05). The findings suggest that both flaxseed- and oatmeal-based gels are promising natural carriers for green tea phytochemicals, offering standardized dosing and potential cognitive health benefits. Further studies are warranted to assess the in vivo bioavailability and long-term stability of these formulations.

## 1. Introduction

In recent years, edible gels have emerged as a highly versatile and consumer-friendly form for delivering bioactive compounds, particularly in the fields of functional foods, nutraceuticals, and pharmaceutical applications [1,2]. Their soft, semi-solid structure allows for easy ingestion without the need for water, making them particularly suitable for populations with swallowing difficulties, such as children and the elderly. Furthermore, edible gels offer the advantage of precise dosing, the homogenous distribution of active ingredients, and the potential for controlled release, which enhances the bioavailability and therapeutic efficacy of the incorporated substances [3,4,5]. The growing demand for clean-label and natural origin health products has driven innovation toward the use of plant-based gelling agents in edible gel formulations [6]. Natural gelling materials such as mucilage or other polysaccharides not only serve as structural components but also align with consumer expectations for recognizable, minimally processed ingredients. These agents correspond to the clean label concept, as they typically require no chemical modification, are free from synthetic additives, and may possess additional nutritional or therapeutic properties. Their use enhances the appeal of the final product, especially in markets prioritising transparency, sustainability, and plant-based solutions [6].

Flaxseed (*Linum usitatissimum* L.) mucilage, a polysaccharide-rich byproduct derived from oilseed processing, presents an eco-friendly and functional option for gel formulation. Located primarily in the seed hull, this mucilage is rich in arabinoxylans, which are capable of forming gel networks through ferulic acid crosslinking, not only contributing to desirable rheological properties but also offering prebiotic potential [7,8,9].

Oats (*Avena sativa* L.) are a rich source of (1→3)(1→4)-β-D-glucans, which are naturally occurring polysaccharides concentrated in the sub-aleurone and endosperm cell walls, known for their high viscosity and ability to form stable gel networks [10]. Recognized by the European Food Safety Authority (EFSA), oat β-glucans are scientifically substantiated to contribute to the maintenance of normal blood LDL-cholesterol levels, improve satiety, and attenuate postprandial glycemic responses [11,12].

Green tea (*Camellia sinensis* (L.) Kuntze) is a medicinal plant rich in polyphenolic compounds, particularly the profile of catechins [13]. These compounds exhibit potent antioxidant, anti-inflammatory, cardioprotective, anticancer, hypoglycemic, antibacterial, antiviral, neuroprotective and anticarcinogenic properties [14]. Traditionally, green tea preparations have been used for the relief of fatigue and sensation of weakness [15]. The unique phytochemical profiles of L-theanine, catechins, and methylxanthine alkaloids together exert synergistic effects on mental performance. Numerous studies have demonstrated that green tea extracts can enhance neuroprotection by reducing oxidative stress, modulating neurotransmitter activity, and improving synaptic plasticity [14,16,17]. Moreover, the moderate caffeine content naturally present in *Camellia sinensis* has been shown to increase alertness, attention, and reaction time, making it particularly valuable for individuals seeking a mental performance boost without excessive stimulation [16]. Including green tea extract in a standardized gel format ensures the consistent dosing of these cognition-enhancing compounds, which is especially relevant in functional food applications targeted at students, professionals, or older adults looking to maintain mental clarity and reduce age-related cognitive decline. However, variability in green tea dosage occurs when it is consumed in traditional forms, such as teas [15]. Incorporating green tea extract into a structured gel matrix enables the standardization of the dose, protection of the active components from environmental degradation, and modulation of their release in the digestive tract, thereby improving efficacy and safety.

The present study investigates the development of natural-origin edible gels containing green tea extract and plant-derived gelling agents, including whole flaxseed, ground flaxseed, and oat flake polysaccharides. The resulting gels hold strong potential across the food, pharmaceutical, and cosmetic industries; they offer a convenient, antioxidant-rich format for health-conscious consumers, they serve as effective carriers for phytochemicals, supporting chronic disease management and enhanced bioavailability, and they even may be developed as nutricosmetics, which are edible beauty products that deliver skin-beneficial compounds, highlighting their versatility, natural origin, and consumer appeal [1]. Despite these promising aspects, several scientific uncertainties remain. There is a lack of comparative data on the biopharmaceutical behavior of green tea compounds within different plant-derived gel matrices, particularly regarding how the matrix composition influences the stability, rheological properties, and release profile of phenolics.

Therefore, this study aims to evaluate the physicochemical and biopharmaceutical properties of natural-origin edible gels incorporating *C. sinensis* extract, using different plant-derived gelling agents with a focus on stability, phenolic release, and potential cognitive health applications.

## 2. Results

**Figure 1:**
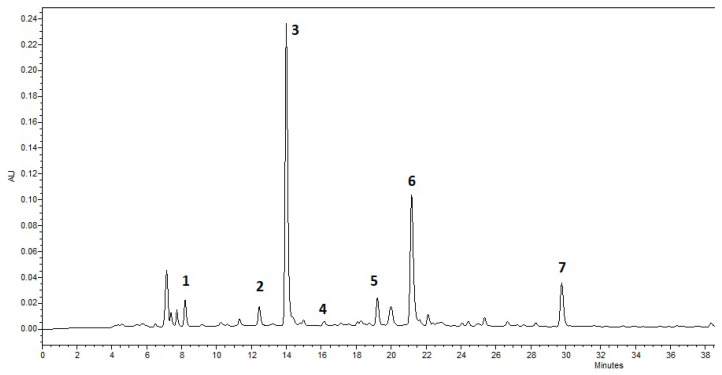
Chromatogram of the green tea extract: 1—gallic acid, 2—epigallocatechin, 3—caffeine, 4—catechin, 5—epicatechin, 6—epigallocatechin-3-gallate, 7—epicatechin-gallate.

The profile of catechins and caffeine was determined in the green tea extract. The identified (Figure 1) compounds were gallic acid, epigallocatechin, caffeine, catechin, epicatechin, epigallocatechin-3-gallate, and epicatechin-gallate.

Their determined amounts are presented in Table 1.

The dominant compound in green tea extract was caffeine, while other compounds were present in lower amounts.

The amount of extract added to the gels was calculated based on the caffeine content as follows: one gel dose (18.9 g) contained dry green tea extract containing 50 mg of caffeine. Accordingly, one dose of gel contained 2.46 mg of gallic acid, 25.80 mg of epigallocatechin, 3.11 mg of catechin, 16.10 mg of epicatechin, 33.66 mg of epigallocatechin-3-gallate, and 10.28 mg of epicatechin-gallate.

NL-1 and ML-1 gels were made from flaxseeds; NL-1 gel used whole seeds, while ML-1 was ground. VA-1 and SA-1 were made from oat flakes. VA-1 flakes were smaller, more finely ground, while SA-1 was coarser. This was conducted to compare the release of gelling agents from flaxseeds and oat flakes, as well as to compare the properties of the resulting gels.

**Figure 2:**
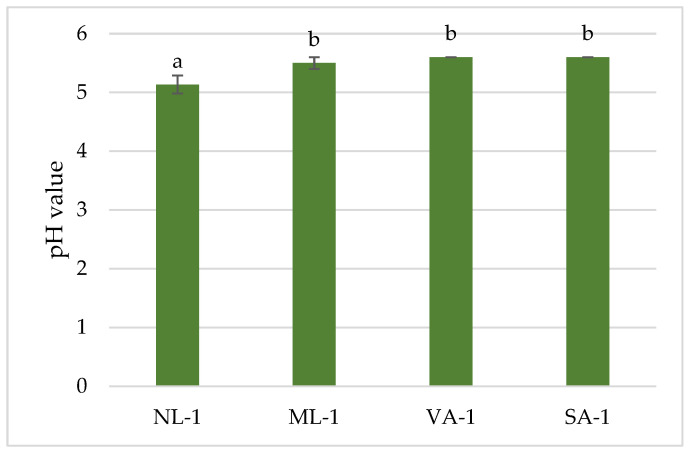
pH values of gels with green tea extract (n = 3). Bars with different letters indicate statistically significant differences between the means (*p* < 0.05).

The pH of gels with green tea extract was slightly acidic (Figure 2). The lowest pH value (5.1 (0.15)) was found for gel NL-1, which was made from unground flaxseeds. The highest pH was found for gels VA-1 (5.6 (0.01)) and SA-1 (5.6 (0.01)). The pH of the NL-1 gel was statistically significantly (*p* < 0.05) lower than that of the remaining gels. However, the pH of all gels was suitable for oral administration.

To assess the quality of gel formulations, the rheological properties of the gels were evaluated, and the consistency coefficient and flow index were calculated (Table 2).

The consistency coefficient shows the shear stress value when the shear rate is 1 s^−1^. This value shows the viscosity of the formulations; he higher the value, the higher the viscosity. The highest consistency coefficient was determined for the gel VA-1—24.33 (2.60) Pa·s, and the lowest was found for the gel NL-1—2.11 (0.37) Pa·s.

A statistically significant difference was found between NL-1 and ML-1 (*p* < 0.05). This indicates that the gelling agents from ground flaxseed can more easily enter water and form a gel with a higher viscosity. When comparing VA-1 and SA-1 gels, it was found that the gel made from medium-sized oat flakes has a statistically significant (*p* < 0.05) higher viscosity.

The flow index shows the affinity of the formulations to Newtonian fluids: the closer the value is to 1, the closer the properties are to Newtonian fluids. The highest flow index was determined for the gel NL-1, 0.47 (0.02),and the lowest for the gel VA-1, 0.31 (0.03). Statistically significant (*p* < 0.05) differences were found between the flow index values of the tested gels NL-1 and ML-1 and between VA-1 and SA-1. This shows that the properties of gels obtained from coarser raw materials were closer to Newtonian fluids.

A dissolution test was performed to evaluate the release of active compounds from the tested gels. After the dissolution test, the total amount of phenolic compounds in the acceptor medium was determined, and the concentrations of the following seven compounds were determined: gallic acid, epigallocatechin, caffeine, catechin, epicatechin, epigallocatechin-3-gallate, and epicatechin-gallate.

**Figure 3:**
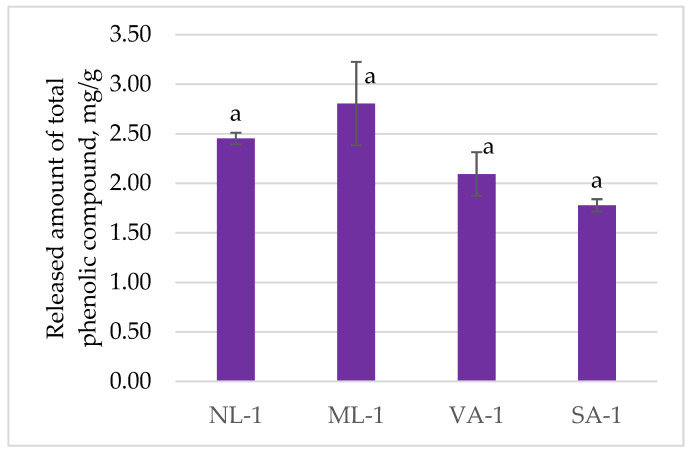
The released amount (mg/g) of total phenolic compounds from gels with green tea extract (n = 3). Bars with different letters indicate statistically significant differences between the means (*p* < 0.05).

The released amounts of total phenolic compounds are presented in Figure 3. The highest amount of total phenolic compounds was released from gel ML-1, 2.81 (0.82) mg, and the lowest from gel SA-1, 1.78 (0.13) mg. After statistical analysis of the results, no statistically significant (*p* > 0.05) differences were found between the formulations; thus, we can state that neither the gelling agent nor its fineness influenced the release of the total amount of phenolic compounds.

**Figure 4:**
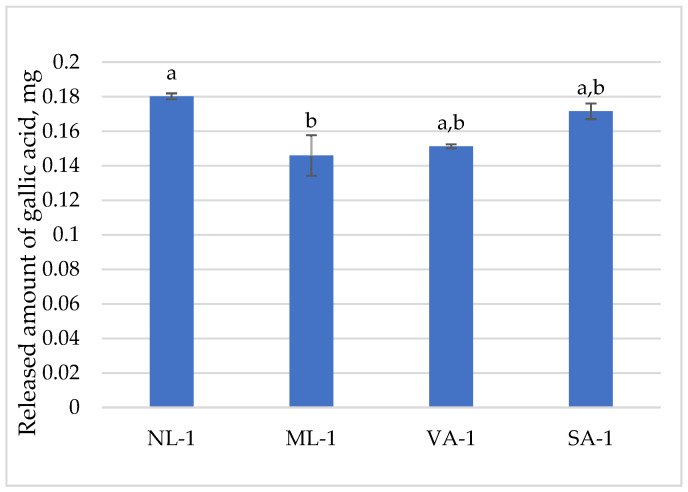
The released amount of gallic acid compounds from gels with green tea extract (n = 3). Bars with different letters indicate statistically significant differences between the means (*p* < 0.05).

The released amount of gallic acid is presented in Figure 4. The amount of gel taken for the dissolution test contained 0.13 mg of gallic acid. The formulation NL–1 released the most gallic acid in one hour, 138%, which was slightly less than SA-1 (131%). The formulations ML-1 (111%) and VA-1 (116%) released the least gallic acid. The amounts of gallic acid released from the different gels were similar; statistically significant differences were observed between NL-1 and ML-1 (*p* < 0.05).

**Figure 5:**
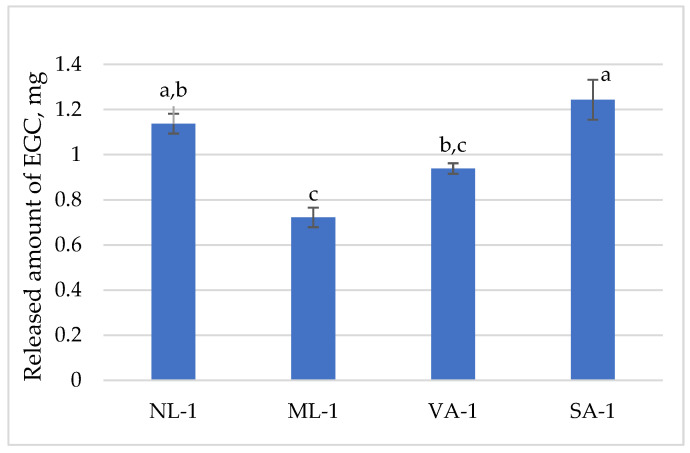
The released amount of epigallocatechin compounds from gels with green tea extract (n = 3). Bars with different letters indicate statistically significant differences between the means (*p* < 0.05).

The released amount of epigallocatechin (EGC) is presented in Figure 5. A gel amount of 1 g contained 1.37 mg of epigallocatechin. It was released best from the gel SA-1 (91%) and least from ML-1 (52%). A statistically significant difference was found between the amounts of epigallocatechin released from NL-1 and ML-1 (*p* < 0.05); thus, a lower amount was released from ground flaxseed than from unground flaxseed gel. Additionally, a statistically significantly lower amount was released from VA-1 gel than from SA-1 (*p* < 0.05).

**Figure 6:**
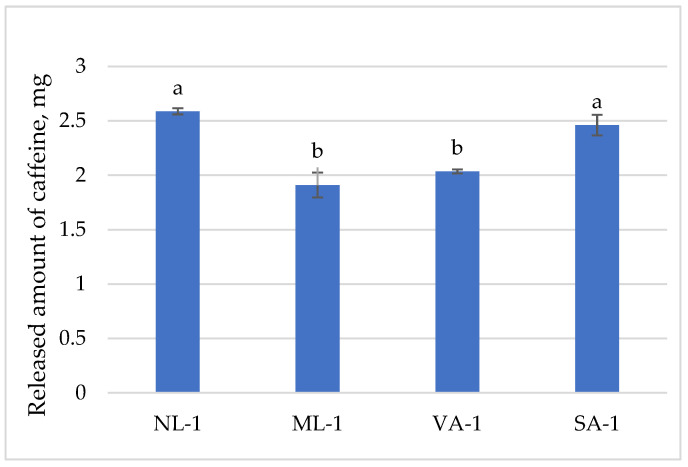
The released amount of caffeine compounds from gels with green tea extract (n = 3). Bars with different letters indicate statistically significant differences between the means (*p* < 0.05).

The released amount of caffeine is presented in Figure 6. An amount of 1 g of the tested gels contained 2.6 mg of caffeine. The results of the studies showed that NL-1 and SA-1 released a slightly higher amount of caffeine than ML-1 and VA-1 (97% and 92%, 72% and 76%, respectively). When comparing gels from flaxseeds, a statistically significant difference was found between NL-1 and ML-1. Gels from different coarseness of oat flakes also differed; a statistically significant difference was found between VA-1 and SA-1.

**Figure 7:**
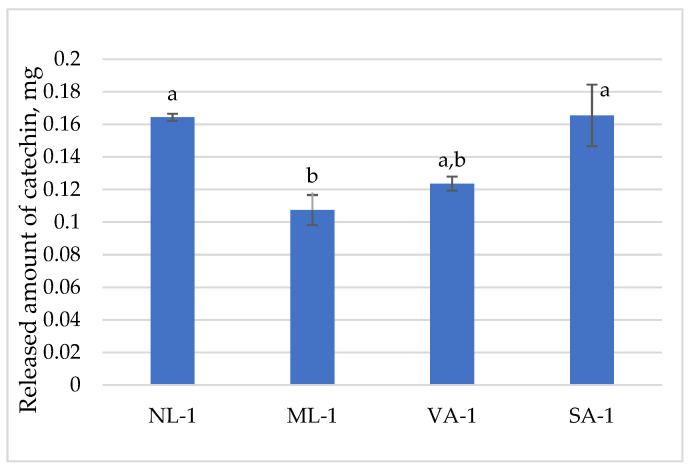
The released amount of catechin from gels with green tea extract (n = 3). Bars with different letters indicate statistically significant differences between the means (*p* < 0.05).

The released amount of catechin is presented in Figure 7. The amount of catechin in the gel taken for the dissolution study was 0.17 mg. The highest amounts of catechin were released from NL-1 gel (99.7%) and SA-1 gel (100%), and the lowest amounts were from ML-1 (65%) and VA-1 (75%). The amount of catechin released was influenced by the coarseness of the flaxseeds; the amount released from ground flaxseed gel was statistically significantly lower than from whole seeds (*p* < 0.05). Meanwhile, the amount released from medium-sized and coarse oat flakes was similar and did not differ significantly (*p* > 0.05).

**Figure 8:**
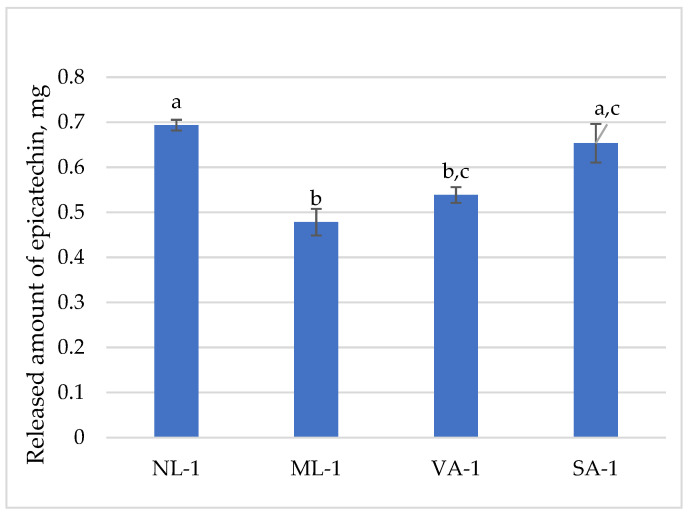
The released amount of epicatechin from gels with green tea extract (n = 3). Bars with different letters indicate statistically significant differences between the means (*p* < 0.05).

Figure 8 presents the released amount of epigallocatechin. One gram of gel taken for the dissolution test contained 1.78 mg of epicatechin. The highest amounts of epicatechin were released from the NL-1 gel (81%) and SA-1 gel (77%), and the lowest amounts were from ML-1 (56%) and VA-1 (63%). The amount of epicatechin released from ground flaxseed gel was statistically significantly lower than from whole seeds (*p* < 0.05). The released amounts of epicatechin from the different oatmeal gels were similar, and no statistically significant differences were found (*p* > 0.05).

**Figure 9:**
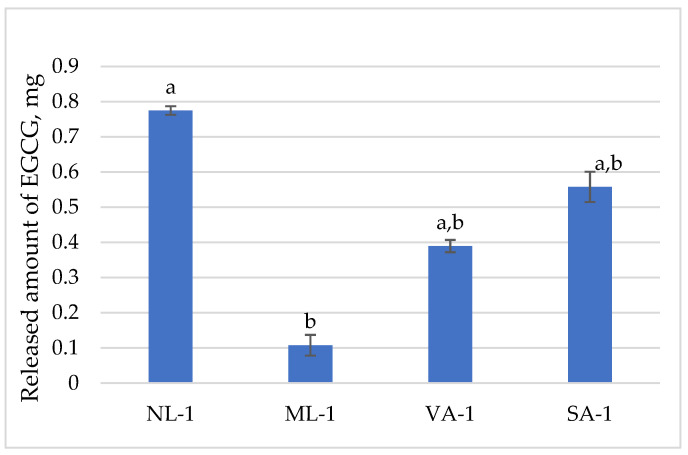
The released amount of epigallocatechin-3-gallate from gels with green tea extract (n = 3). Bars with different letters indicate statistically significant differences between the means (*p* < 0.05).

The released amount of epigallocatechin-3-gallate is presented in Figure 9. An amount of 1 g of gel taken for the dissolution test contained 1.78 mg of epigallocatechin-3-gallate. The highest amounts of epigallocatechin-3-gallate were released from NL-1 gel (43%) and SA-1 gel (31%), and the lowest amounts were from ML-1 (6%) and VA-1 (21%). The released amount of epigallocatechin-3-gallate was statistically significantly higher from NL-1 than from ML-1 (*p* < 0.05). No statistically significant differences were found between the other formulations (*p* > 0.05).

**Figure 10:**
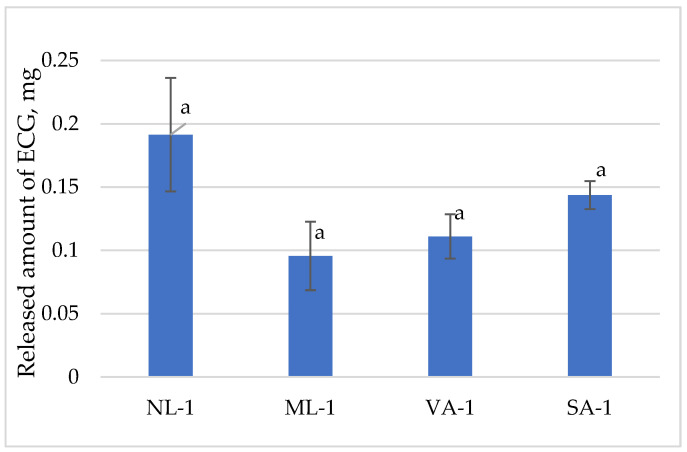
The released amount of epicatechin-gallate from gels with green tea extract (n = 3). Bars with different letters indicate statistically significant differences between the means (*p* < 0.05).

Figure 10 presents the released amount of epicatechin-gallate. One gram of gel taken contained 1.78 mg of epicatechin-gallate. The highest amounts of epicatechin-gallate were from the NL-1 gel (35%) and SA-1 gel (26%), and the lowest amounts were from ML-1 (18%) and VA-1 (20%). The released amounts of epicatechin-gallate from the different gels were similar, and no statistically significant differences were found (*p* > 0.05).

## 3. Discussion

Understanding the phytochemical composition of green tea is essential for developing effective delivery systems, as its health benefits depend on the presence and stability of key bioactive compounds. This study focuses on how these compounds behave within natural-origin edible gels, aiming to enhance both their stability and release profiles. To our knowledge, this is the first study comparing the release behavior of green tea phenolics from gels formulated with unmodified flaxseeds and oat flakes of different particle sizes. Conventional hydrogels rely on extracted or purified polymers and mainly edible coatings [18,19,20,21,22]. In contrast, our approach utilizes whole or minimally processed plant materials. This clean-label strategy aligns with current consumer trends toward natural, familiar, and sustainable ingredients, making the gels highly attractive for the development of functional foods and nutraceuticals [1,23]. Furthermore, the direct use of whole flaxseeds and coarse or fine oat flakes remains largely unexplored in the scientific literature. These plant matrices have dual functionality, as they provide both structural and bioactive approaches to phytochemical delivery [23]. Notably, the formulation process requires no chemical additives or sophisticated equipment, making it readily adaptable for small-scale production or industrial scaling [24,25].

The phytochemical composition of green tea contains several classes of compounds, with phenolics, alkaloids, and amino acids being of the most importance [14]. Catechin origin compounds tend to be the most antioxidant active. Furthermore, various studies identify them as key compounds for the anticancer, antidiabetic, cardiovascular, and neuroprotective effects [26,27,28]. Green tea has also been found to reduce anxiety and to have a positive impact on memory and concentration. However, it has been found that individual purified compounds do not provide the most potent effect but rather a complex of compounds [16]. The catechins most commonly found in green tea are classified into simple catechins (catechin, epicatechin, gallocatechin, epigallocatechin) and ester-catechins (epigallocatechin gallate, epicatechin gallate, gallocatechin gallate) [29]. Ester-catechins can be broken down into simple catechins and gallic acid. Studies suggest that catechins form up to 80% of the total phenolics that are present in the brewed tea and up to 40% of the dry weight of green tea [27,28]. The amount of gallic acid in green tea products is known to vary depending on processing methods, with higher levels often observed due to the hydrolysis of catechin gallates during processing [26]. After evaluating the phenolic compound profile of green tea extract, seven phenolic compounds were identified, with caffeine dominating the most, with a concentration of 57.05 (1.03) mg/g. Similar results were obtained by other researchers studying green tea extracts. They identified gallic acid, epigallocatechin, epigallocatechin-3-gallate, and epicatechin [27,30]. Furthermore, catechins, gallic acid, and methylxanthines are key markers for distinguishing green tea samples based on their geographical origin and production methods. However, broader studies incorporating diverse samples, manufacturing techniques, and seasonal variations are needed to fully understand the factors influencing their composition [26]. This variability highlights the need for the standardization of green tea extracts in functional formulations to ensure consistent bioactive delivery and reproducible health effects.

Gels are semi-solid preparations that consist of liquids gelled by a suitable gelling agent (1). Although gels are most often used on the skin, they are increasingly being adapted for oral use. Edible gels are an excellent alternative for delivering bioactive compounds [15,22]. Solid dosage forms (such as capsules and tablets) are easy to manufacture and have many other advantages, but they are not suitable for people with swallowing difficulties and are also not convenient to take because they need to be taken with water (4). The following natural and nutrient-rich gelling agents were chosen as the bases for the gels: flaxseeds (*Linum usitatissimum* L.) and oatmeal (*Avena sativa* L.). Oats (*Avena sativa* L.) are well recognized for their nutritional and functional components, particularly β-glucans, phenolic acids (such as ferulic and caffeic acids), and unique proteins like avenins and globulins, which contribute to both health benefits and technological properties of oat-based products [10,31,32]. Extracting proteins and other bioactive substances from oats is challenging due to the structural complexity of whole oat grains and the tight association of β-glucans with cell wall components [31,33]; therefore, oat flakes were selected in this study as a more practical source of gelling agents. Upon heating, the main oat protein, globulin, denatures and breaks down into monomers, promoting aggregation and forming intermolecular bonds that stabilize the gel network [10,34]. This mechanism supports the gel’s structural integrity and influences the release of the incorporated bioactive ingredient. Although oats are rich in β-glucans and phenolic compounds, their release depends strongly on the processing method and matrix structure [35,36]. Research on natural gels is restricted by the lack of standardization of the raw materials from which the gels are made. There is a lack of scientific research to assess the differences caused by differences in both plant raw material and its processing [37]. In our study, the total phenolic release from coarse oat flake gels (SA-1) was slightly higher than from finer oat flake gels (VA-1), as shown in Figure 3. This can be attributed to the more open gel network in SA-1, which allows for more efficient diffusion of phenolics from the incorporated green tea extract. Interestingly, while the oat-based gels were not expected to contribute significant amounts of their phenolics (due to minimal extraction of oat compounds under the tested conditions), the matrix still played a crucial role in modulating the release kinetics of the green tea components. These findings correspond with previous studies suggesting that the structural properties of β-glucan-rich matrices can influence both gel viscosity and the diffusion of active compounds [36], with coarser matrices often facilitating faster release [22]. Thus, oat-based gels not only provide structural benefits but may also impact the delivery efficiency of phytochemicals.

When flaxseeds are mixed in water, they release mucus, which contains L-arabinose, D-xylose, and D-galactose, while the acidic fraction has L-rhamnose, L-fucose, L-galactose, and D-galacturonic acid [7]. Flaxseeds and their mucilage can be used as a gelling, foaming, encapsulating, emulsifying, suspending, film–forming agent [7]. On the other hand, flaxseeds are not only a source of mucilage, but they are also rich in a variety of bioactive compounds, including lignans (such as secoisolariciresinol diglucoside), omega-3 fatty acids (primarily α-linolenic acid), proteins, and phenolic compounds, which together contribute to their antioxidant, anti-inflammatory, and cardioprotective effects [7,8,9,38]. The phenolic compounds detected in flaxseed matrices are hydroxycinnamic acids, particularly ferulic acid and coumaric acid derivatives [39]. Grinding flaxseeds disrupts the hard outer seed coat, significantly enhancing the release and bioavailability of these compounds, especially phenolics and lignans, which are otherwise poorly accessible in whole seeds [9,10]. In this study, the ML-1 gel containing ground flaxseed exhibited a higher total phenolic release compared to the NL-1 gel with whole flaxseeds, as shown in Figure 3. This suggests that the mechanical breakdown of the seed coat in ML-1 improved the liberation of internal phenolic-rich fractions into the gel matrix, corroborating previous findings that grinding flaxseeds can markedly increase the extractability of phenolic antioxidants [10,11]. This also agrees with the observed rheological data, where the denser network of the ground flaxseed gel may have contributed to the release peculiarities of certain compounds. Moreover, the additional phenolic content originating from the ground flaxseed itself likely contributed to the elevated total phenolic release, indicating that such gels deliver not only the incorporated green tea extract but also the bioactive compounds from the gelling material, enhancing the nutraceutical value of the formulation.

The dissolution test showed that distinct compounds are released differently. The most released are gallic acid (111–138%), catechin (65–100%), caffeine (72–97%), epicatechin (56–81%), and epigallocatechin (52–91%), and the least released are epigallocatechin-3-gallate (6–73%) and epicatechin-gallate (18–35%). Therefore, after the release tests, a higher amount of gallic acid was determined than was in the extract. Different phenolic compounds showed varied release profiles, with gallic acid and catechin released in the highest percentages, likely due to the hydrolysis of ester catechins. In contrast, epigallocatechin-3-gallate and epicatechin-gallate showed the lowest releases, reflecting their larger molecular size and tendency to remain trapped within the gel matrix [30].

When comparing the released amounts of individual compounds from different formulations, a tendency was observed that slightly higher amounts were released from gels NL-1 and SA-1 than from ML-1 and VA-1. Interestingly, NL-1 and SA-1 gels were made from whole flaxseeds and large oat flakes, and their consistency coefficients were lower than ML-1 and VA-1. Thus, we can assume that more gelling compounds are released from more finely ground seeds, which bind the active compounds and prolong their release [7,22]. Incorporating green tea extract into edible gels provides a reproducible platform for delivering defined doses of catechins and gallic acid, thereby addressing the variability commonly found in traditional tea infusions [22]. Recent studies have highlighted that polysaccharide matrix gels enhance the stability and bioavailability of phenolics [3].

Natural gelling agents (such as flaxseeds and oatmeal) can not only be useful as nutraceuticals, but they can also stabilize the incorporated active compounds and improve their bioavailability [33]. Future studies should investigate how optimizing gel formulation parameters can further enhance stability, bioavailability, and consumer acceptability. Furthermore, in vivo studies are crucial for evaluating the actual bioavailability, absorption dynamics, and physiological effects of these formulations under biological conditions.

## 4. Materials and Methods

### 4.1. Materials

The following analytical-grade solvents were used in this study: acetonitrile, methanol, obtained from Sigma-Aldrich (Steinheim, Germany), and 99.8% trifluoroacetic acid from Merck (Darmstadt, Germany). The water was purified using a Millipore Milli-Q apparatus. Epicatechin, catechin, gallic acid, caffeine, (−)-epigallocatechin (EGC), (−)-epicatechin gallate (ECG), (−)-epigallocatechin gallate (EGCG), sodium carbonate, and Folin–Ciocalteu reagent were obtained from Sigma-Aldrich.

**Figure 11:**
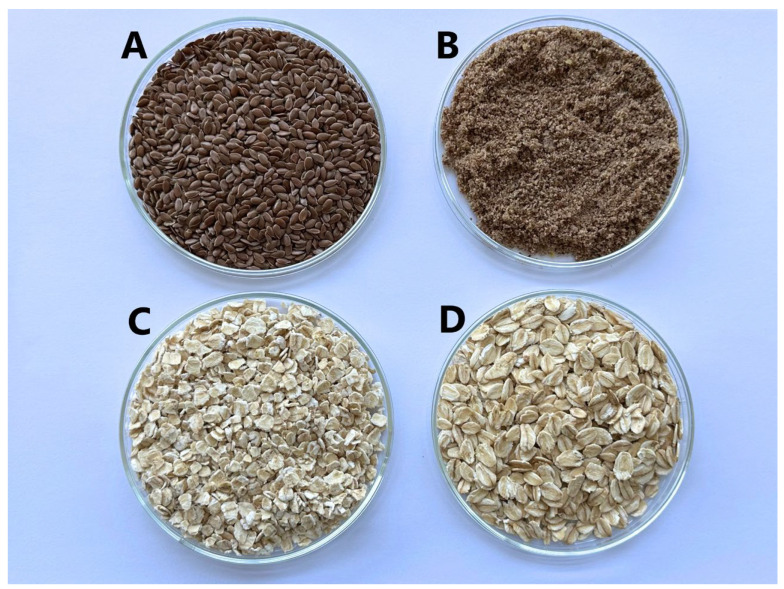
Selected gelling agents: (**A**) whole flaxseeds, (**B**) ground flaxseeds, (**C**) medium-size oatmeal, (**D**) coarse oatmeal.

Green tea (Twinings Gunpowder Green) was obtained from Twinings (Poland) (origin: China). Flaxseeds were from A. Karvelio terapijos-fitoterapijos įmonė (Lithuania) (origin: Kazachstan). Medium-sized oatmeal was obtained from Livinn (Lithuania) (origin: Germany), while coarse oatmeal was from Rapunzel Naturkost (Germany) (Figure 11).

### 4.2. Methods

#### 4.2.1. Preparation of Dry Green Tea Extract

The green tea leaves were milled with an electric grinder. The resulting powder was sieved through a sieve, ensuring a particle diameter of no more than 450 µm. The 50 g of raw material (precise weight) was weighed into 1000 mL flasks, and 500 mL of purified water was added. Extraction was carried out in a water bath with a reflux condenser for 30 min at a temperature of 70 °C. The extract was cooled and centrifuged for 10 min at a speed of 3000 rpm. After centrifugation, the extracts were filtered through cotton wool. Lyophilization was carried out with a Lyophilizer (Telstar LuoQuest, Spain). First, freezing was carried out for 24 h at a temperature of −60 °C; then, drying (lyophilization) was carried out for 2 days at a pressure of 0.1 mBar. The drying rate was measured as 6.61 percent. The lyophilized powder was stored in sealed containers in a dark place until further analysis.

#### 4.2.2. Development of Experimental Edible Gels

Edible gels were prepared using 7 g of raw material and 50 mL of purified water (Table 3). Gels were prepared in heat-resistant chemical beakers on a magnetic stirrer with a heating function, set at 500 rph. The gel was heated to a temperature of 80–90 °C. Heating was carried out until the product gelified (gel is formed).

The prepared gels were cooled and centrifuged at 3000 rpm for 20 min at 20 °C. After centrifugation, the gel layer was separated from the oat and flaxseeds. The gels were stored in a refrigerator at a temperature of 5 ± 3 °C.

Dry green tea extract was added to different types of gel matrices. The amount of green tea extract added was calculated based on the amount of gel produced to achieve a 4.64 percent extract concentration in the final formulation.

#### 4.2.3. High-Performance Liquid Chromatography Assay for the Determination of Individual Chemical Compounds

The HPLC analysis was performed using a “Waters e2695 Alliance system” (Waters, Milford, MA, USA) equipped with a photodiode array detector “Waters 2998”. Separation of phytocompounds was performed using an “ACE Super C18” (ACT, UK) column (C18, 250 mm × 4.6 mm, particle size 3 μm) at 15 °C. The mobile phase consisted of eluent A (0.05% trifluoracetic acid) and B (acetonitrile). The gradient was 0 min., 85% A; 0–30 min., 70% A; 30–50 min., 40% A; 50–56 min., 10% A; 56–65 min., 15% A. Eluent flow rate was 0.5 mL/min, and injection volume was 10 μL. Chromatographic peak identification was carried out according to the analyte and reference compound retention time as well as by comparing their UV absorption spectra at 200–400 nm. The quantification of phytocompounds was carried out using external standard calibration curves. Standard substances were injected at five different concentrations to construct calibration curves by plotting peak area against concentration. The linearity of each calibration curve was confirmed with determination coefficients (R^2^) greater than 0.999. Sample concentrations were calculated from the corresponding calibration equations. The spectra of the standard substances and representative spectra from the analyzed samples are provided in the Supplementary Material (Figures S1–S7). All samples were filtered through a 0.22 μm pore size filter before analysis.

#### 4.2.4. Determination of Total Phenolic Compounds by Spectrophotometric Method

The total phenolic content was determined using the Folin–Ciocalteu method by Slinkard and Singleton 1977 and was slightly modified by Kaunaite et al., 2022 [40,41]. Briefly, 20 μL of the extract was mixed with 5 mL of Folin–Ciocalteau reagent (10-fold diluted) and 4 mL of 7.5% sodium carbonate. Absorbance was measured after one hour at 765 nm. Total phenolic compounds were expressed as gallic acid equivalents (GAE) per gram of raw material and calculated according to the following formula:

GAE = c × V/m, mg/g

where c is gallic acid concentration in mg/mL from the calibration curve; V is the volume in mL; m is the exact weight of the dry material, g.

#### 4.2.5. Determination of pH Value

The pH values of the experimental gels were determined using a pH meter. Before each pH measurement, the electrode was rinsed with purified water and only then immersed in the sample, and the pH indicator was measured. During the study, the electrode was completely immersed in the sample contents.

#### 4.2.6. Determination of the Flow Curve of Gels

Edible gels were analyzed by performing a flow curve study using a rheometer with a plate–cone system with a diameter of 50 mm, an angle of 2 degrees, and a sample thickness of 207 µm. The shear rate was increased from 0.1 to 100 s^−1^. The rheometer was set to room temperature, 23 ± 0.5 °C. The data were processed using the Rheocompass program. The mathematical Power law slope model was used to calculate the consistency coefficient (K) and flow index (n).

#### 4.2.7. Dissolution Test

The dissolution study of experimental edible gels from natural raw materials with green tea extract was carried out using a magnetic stirrer with a heated surface. Purified water was chosen as the acceptor component. An amount of 15 mL of purified water and 1 g of room temperature test gel were poured into the test tubes. A magnetic rod was placed in the test tube.

During the study, the temperature in the test tubes was maintained at 37 ± 0.5 °C, and a 500 rph was set. The study was carried out for an hour, after which the samples were collected and cooled.

After the release study, the samples were analyzed using high-performance liquid chromatography and Folin–Ciocalteu spectrophotometric methods.

#### 4.2.8. Statistical Data Analysis

Data analysis was performed using the “Microsoft Office Excel 2016” and “IBM SPSS statistics 29” computer programs. Tests with edible gels of different compositions were repeated 3 times. The results are presented in the work as the mean (standard deviation). One-way analysis of variance was performed by ANOVA test. Tukey HSD multiple comparison test was used to determine significant differences between means. Data are considered statistically significant when *p* < 0.05.

## 5. Conclusions

The edible gels were formulated from the natural-origin gelling agents flaxseeds, oat flakes, and *Camellia sinensis* (L.) Kuntze extract as a bioactive ingredient. All gel formulations exhibited slightly acidic pH and shear-thinning behavior, with oat-based gels showing higher viscosity. Despite differences in gel consistency and gelling agent type, the in vitro release of total phenolic compounds was comparable across formulations, except for a significantly higher release of epigallocatechin from coarse oatmeal gels. These findings highlight the potential of natural edible gels as effective carriers for green tea phytochemicals, offering a consumer-friendly format for delivering standardized doses of bioactive compounds. Further research is needed to evaluate in vivo bioavailability, sensory properties, and long-term stability to advance their application in functional food or nutraceutical products.

## Figures and Tables

**Table 1:** Content of the phenolic compounds in green tea extract.

Compound	Retention Time	Amount (mg/g (SD))
Gallic acid	8.199	2.81 (0.31)
Epigallocatechin	12.447	29.42 (1.5)
Caffeine	13.997	57.05 (1.03)
Catechin	16.164	3.55 (0.25)
Epicatechin	19.215	18.37 (0.67)
Epigallocatechin–3–gallate	21.174	38.39 (1.72)
Epicatechin–gallate	29.774	11.72 (0.69)

**Table 2:** Rheological properties of gels with green tea extract (n = 3). Different letters indicate statistically significant differences between the means (*p* < 0.05).

Formulation	Consistency Coefficient, Pa·s	Flow Index
NL-1	2.11 (0.37) ^a^	0.47 (0.02) ^a^
ML-1	8.61 (0.54) ^b^	0.35 (0.01) ^b^
VA-1	24.33 (2.60) ^c^	0.31 (0.03) ^b^
SA-1	9.62 (2.21) ^b^	0.44 (0.04) ^a^

**Table 3:** Compositions of tested gels.

Formulation	Gelling Agent	Amount of Gelling Agent	Amount of Purified Water (mL)	Amount of Green Tea Dry Extract (%)
NL-1	Whole flaxseeds	7.0	50.0	4.64
ML-1	Ground flaxseeds	7.0	50.0	4.64
VA-1	Medium-size oatmeal	7.0	50.0	4.64
SA-1	Coarse oatmeal	7.0	50.0	4.64

## Data Availability

Data are available in a publicly accessible repository.

## Supplementary Materials

The following supporting information can be downloaded at: https://www.mdpi.com/article/10.3390/molecules30132789/s1, Figure S1: Chromatogram and diode array detector (DAD) UV spectra of gallic acid: (A) chromatogram of the gallic acid standard substance; (B) spectrum of the gallic acid standard substance; (C) representative spectra of gallic acid detected in the analysed samples; Figure S2: Chromatogram and diode array detector (DAD) UV spectra of EGC: (A) chromatogram of the EGC standard substance; (B) spectrum of the EGC standard substance; (C) representative spectra of EGC detected in the analysed samples; Figure S3: Chromatogram and diode array detector (DAD) UV spectra of caffeine: (A) chromatogram of the caffeine standard substance; (B) spectrum of the caffeine standard substance; (C) representative spectra of caffeine detected in the analysed samples; Figure S4: Chromatogram and diode array detector (DAD) UV spectra of catechin: (A) chromatogram of the catechin standard substance; (B) spectrum of the catechin standard substance; (C) representative spectra of catechin detected in the analysed samples; Figure S5: Chromatogram and diode array detector (DAD) UV spectra of epicatechin: (A) chromatogram of the epicatechin standard substance; (B) spectrum of the epicatechin standard substance; (C) representative spectra of epicatechin detected in the analysed samples; Figure S6: Chromatogram and diode array detector (DAD) UV spectra of EGCG: (A) chromatogram of the EGCG standard substance; (B) spectrum of the EGCG standard substance; (B) representative spectra of EGCG detected in the analysed samples; Figure S7: Chromatogram and diode array detector (DAD) UV spectra of ECG: (A) chromatogram of the ECG standard substance; (B) spectrum of the ECG standard substance; (C) representative spectra of ECG detected in the analysed samples.
