# Supplementary material for: Natural-Origin Edible Gels as Delivery Systems for Green Tea Extract: Formulation, Physicochemical, and Biopharmaceutic Profile Assessment
Source: Molecules. 2025 Jun 28;30(13):2789. doi: 10.3390/molecules30132789 (PMC12250661; doi:10.3390/molecules30132789)

Figure S1. Chromatogram and diode array detector (DAD) UV spectra of gallic acid: (A) chromatogram of the gallic acid standard substance; (B) spectrum of the gallic acid standard substance; (C) representative spectra of gallic acid detected in the analysed samples.

A

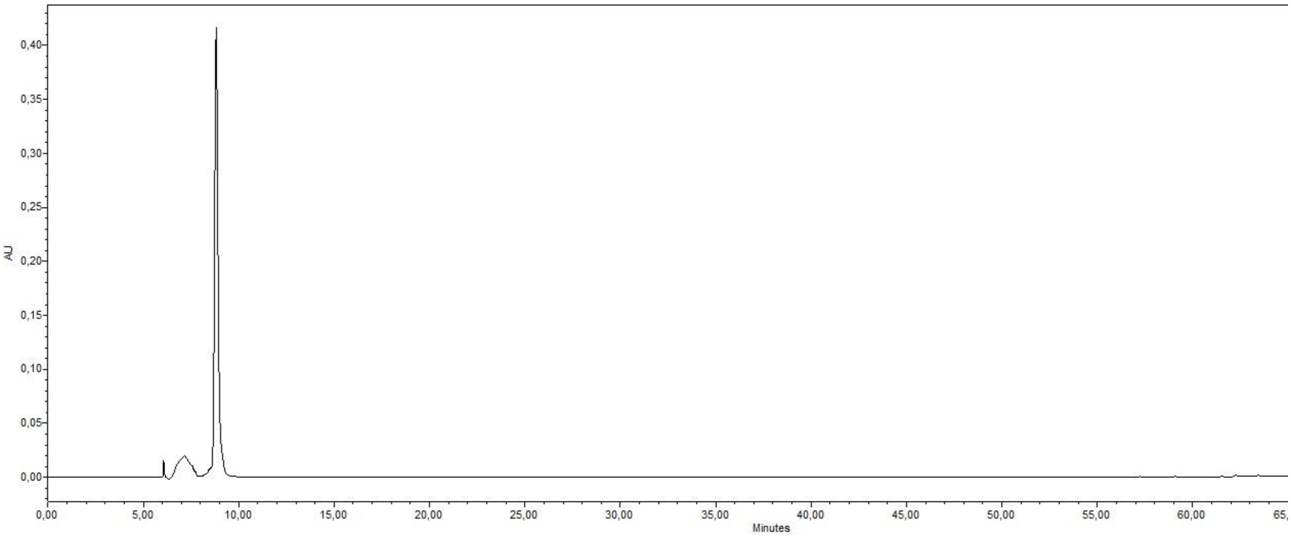

B

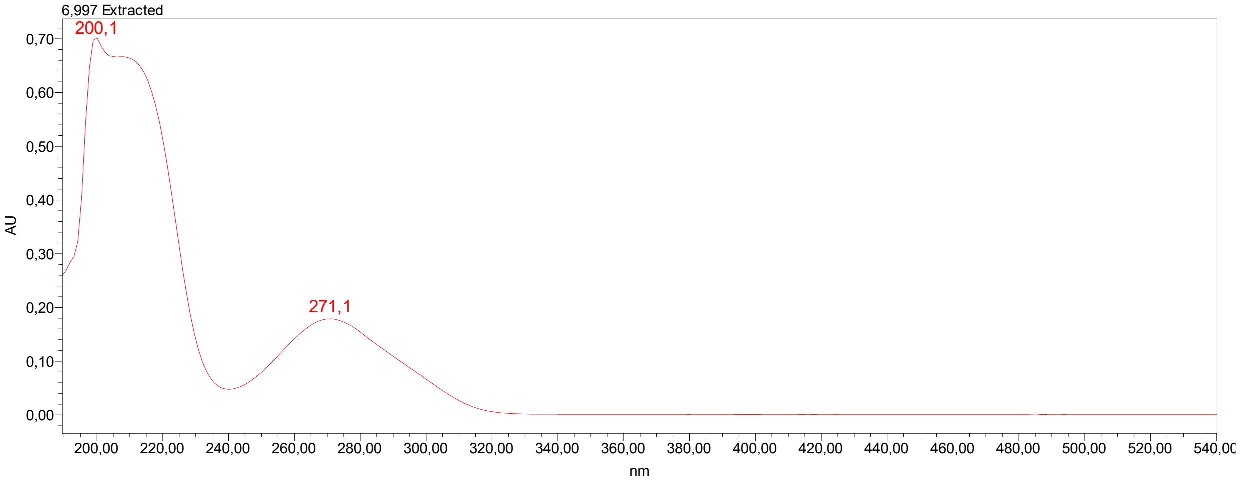

C

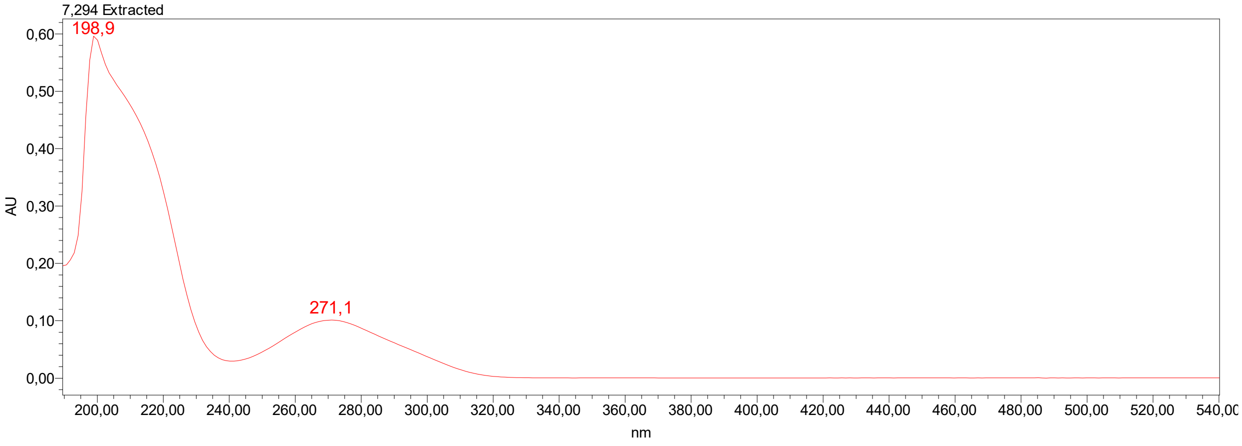

Figure S2. Chromatogram and diode array detector (DAD) UV spectra of EGC: (A) chromatogram of the EGC standard substance; (B) spectrum of the EGC standard substance; (C) representative spectra of EGC detected in the analysed samples.

A

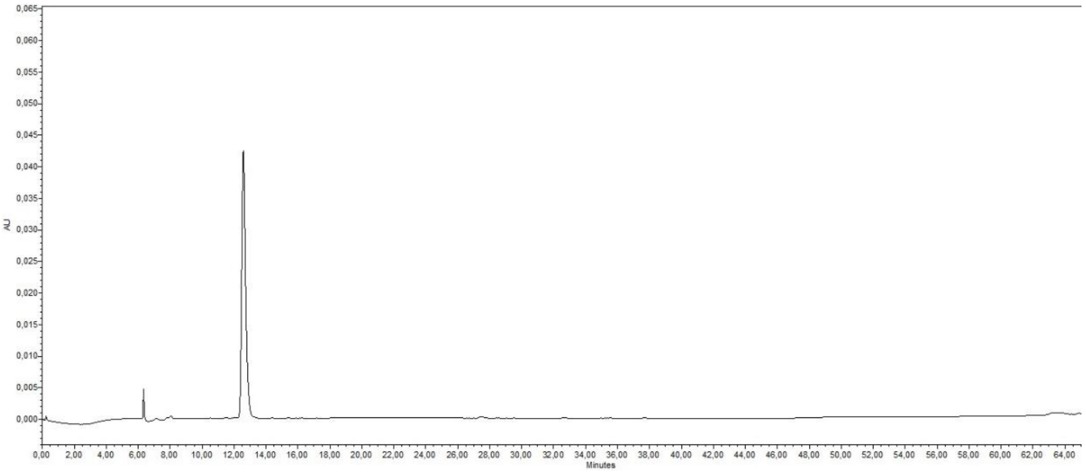

B

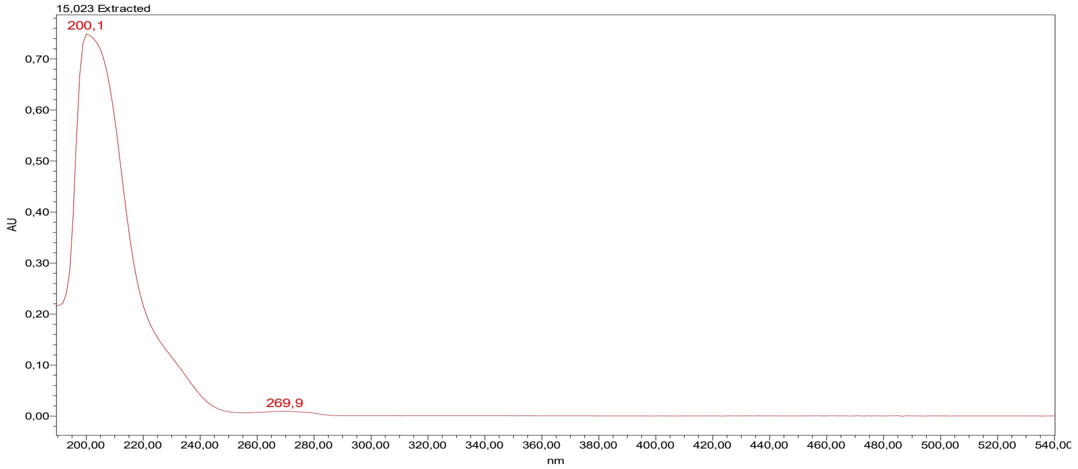

C

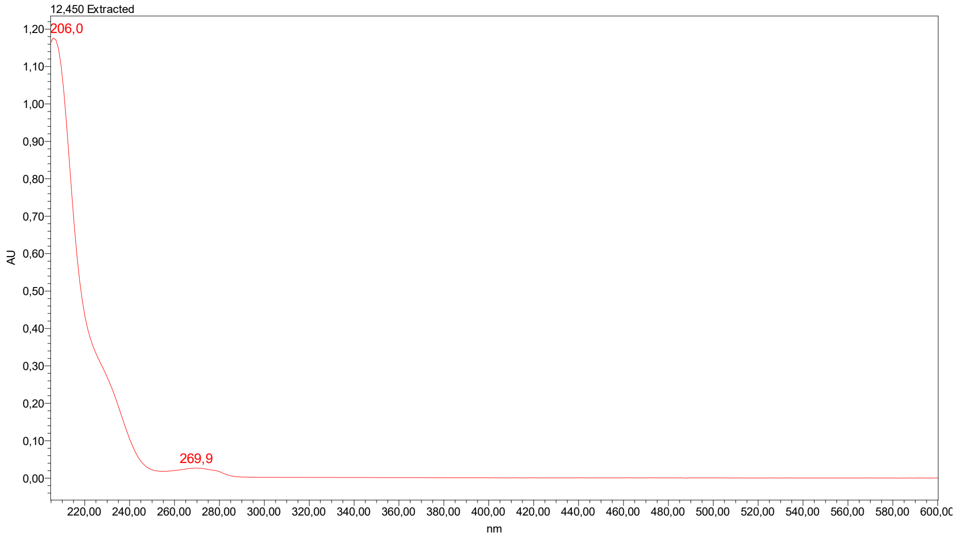

Figure S3. Chromatogram and diode array detector (DAD) UV spectra of caffeine: (A) chromatogram of the caffeine standard substance; (B) spectrum of the caffeine standard substance; (C) representative spectra of caffeine detected in the analysed samples.

A

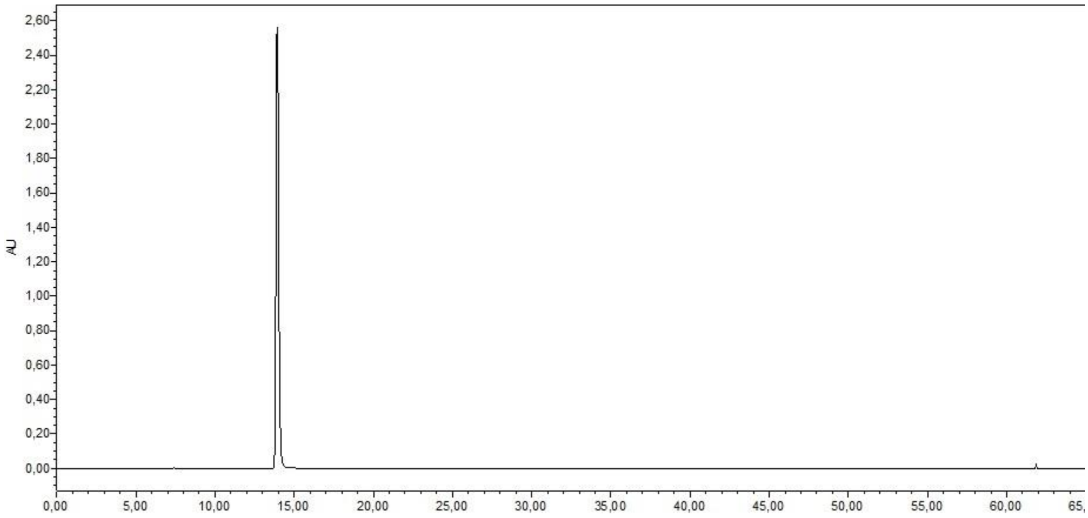

B

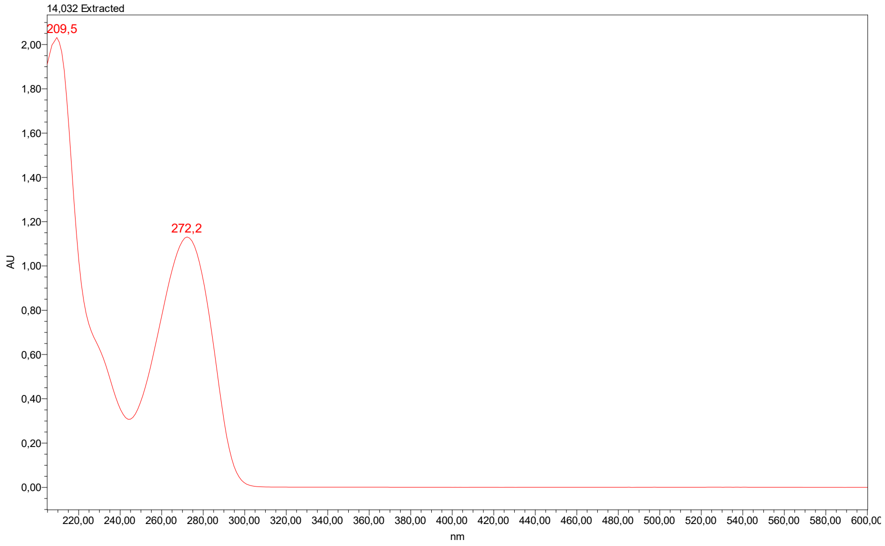

C

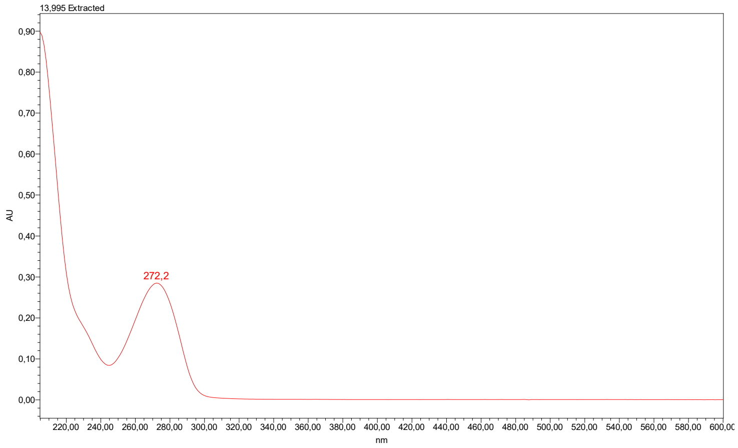

Figure S4. Chromatogram and diode array detector (DAD) UV spectra of catechin: (A) chromatogram of the catechin standard substance; (B) spectrum of the catechin standard substance; (C) representative spectra of catechin detected in the analysed samples.

A

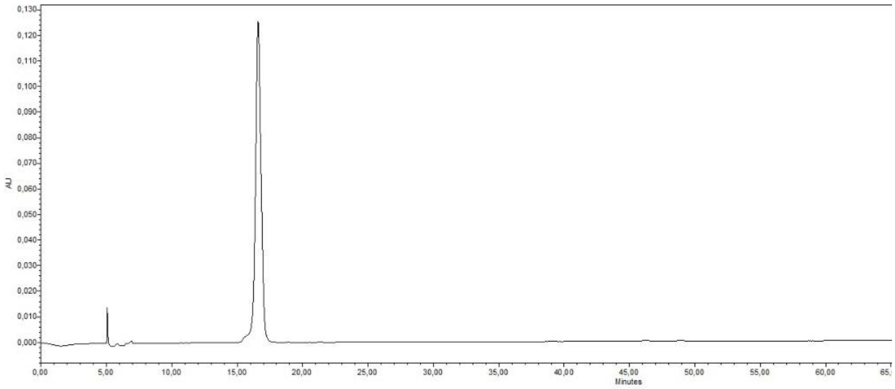

B

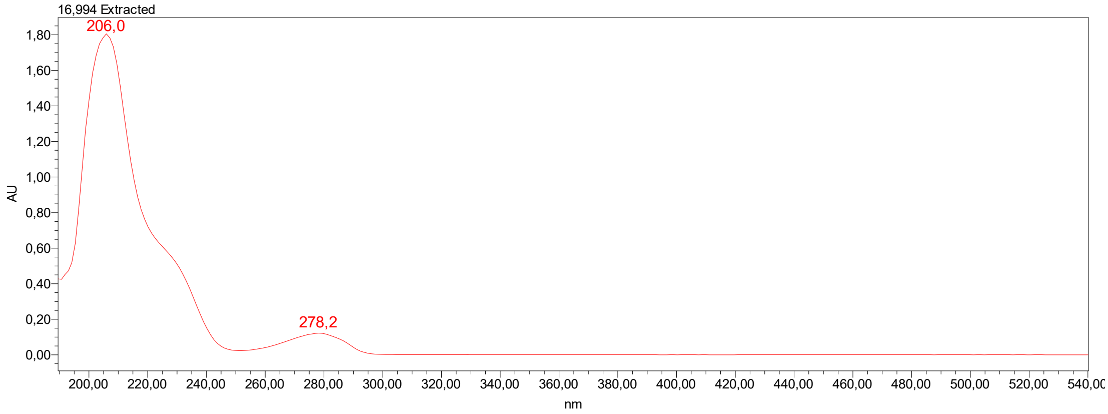

C

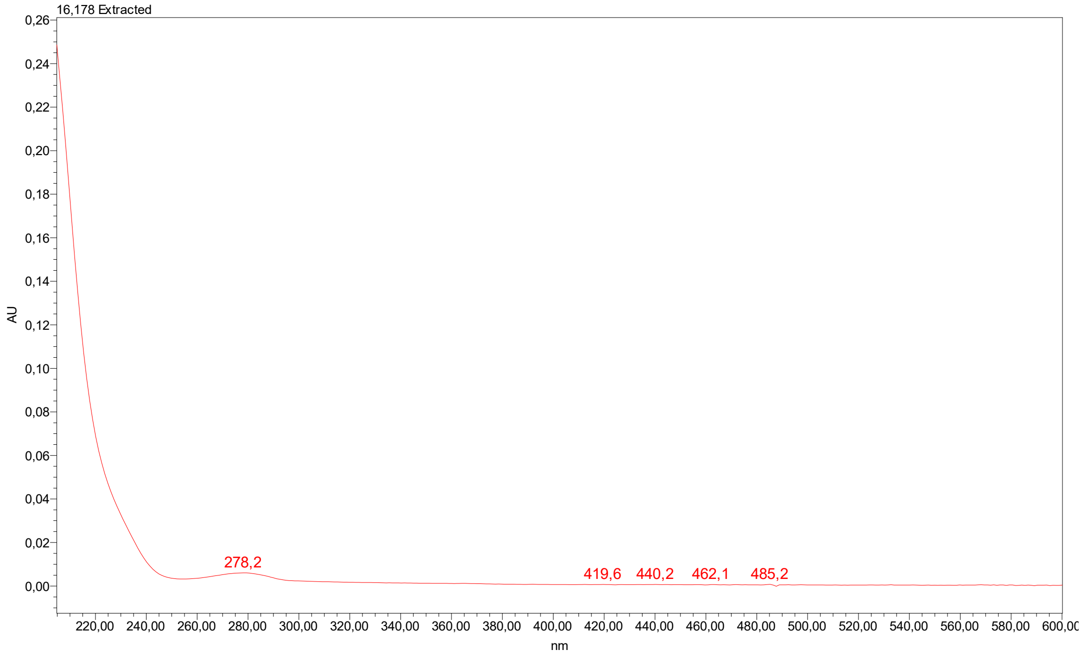

Figure S5. Chromatogram and diode array detector (DAD) UV spectra of epicatechin: (A) chromatogram of the epicatechin standard substance; (B) spectrum of the epicatechin standard substance; (C) representative spectra of epicatechin detected in the analysed samples.

A

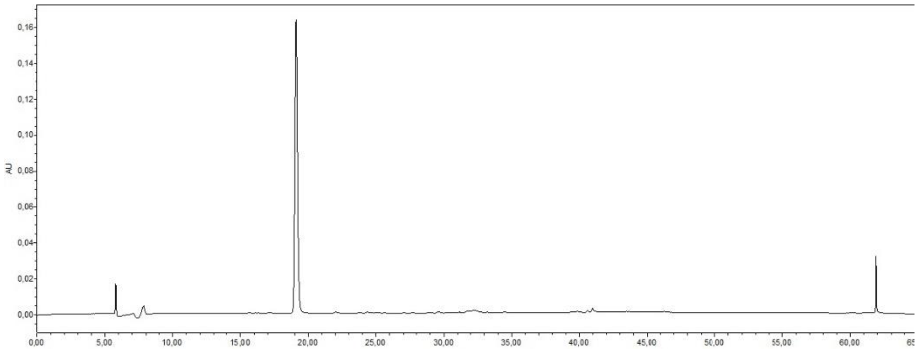

B

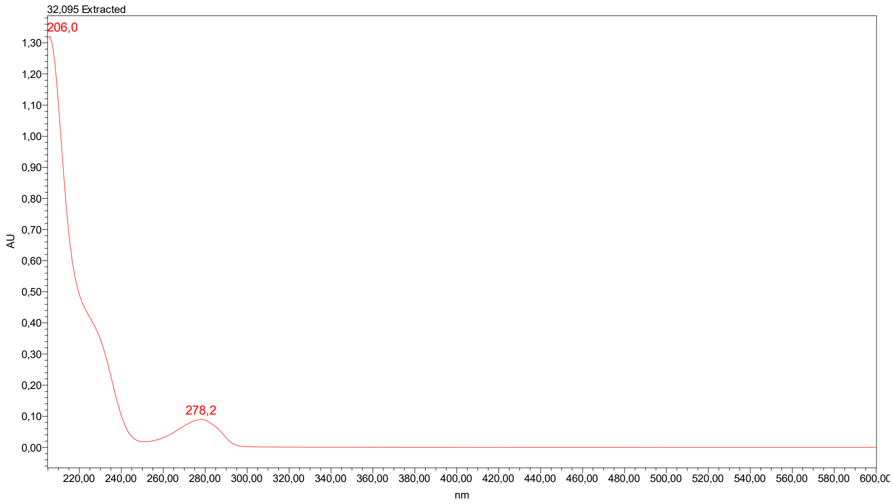

B

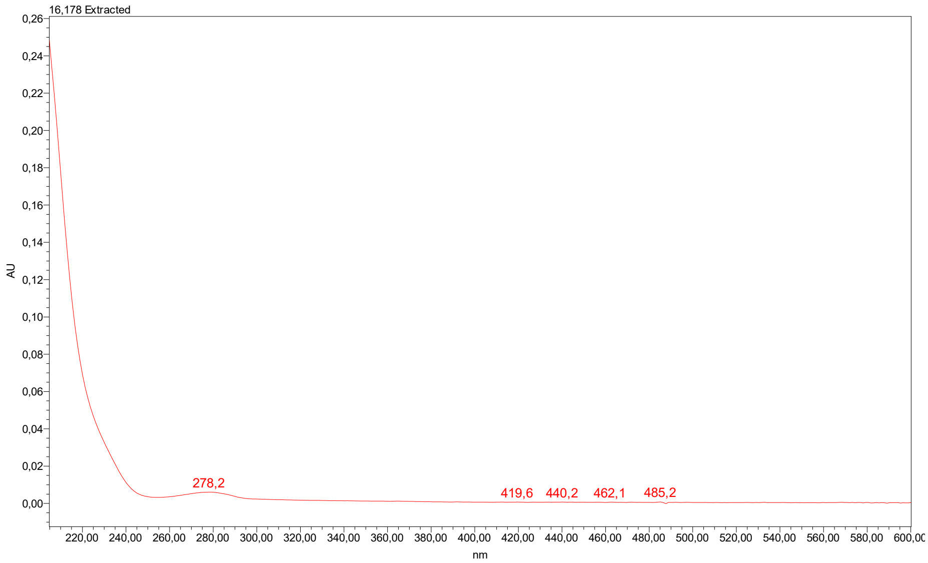

Figure S6. Chromatogram and diode array detector (DAD) UV spectra of EGCG: (A) chromatogram of the EGCG standard substance; (B) spectrum of the EGCG standard substance; (B) representative spectra of EGCG detected in the analysed samples.

A

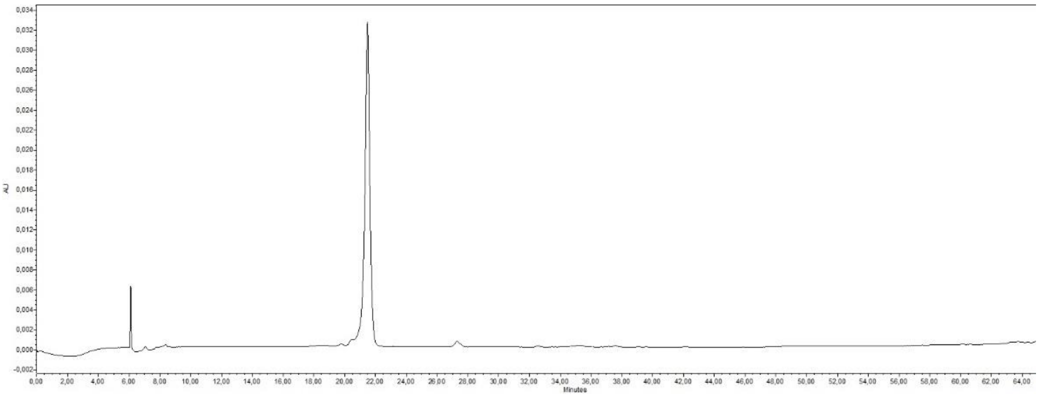

B

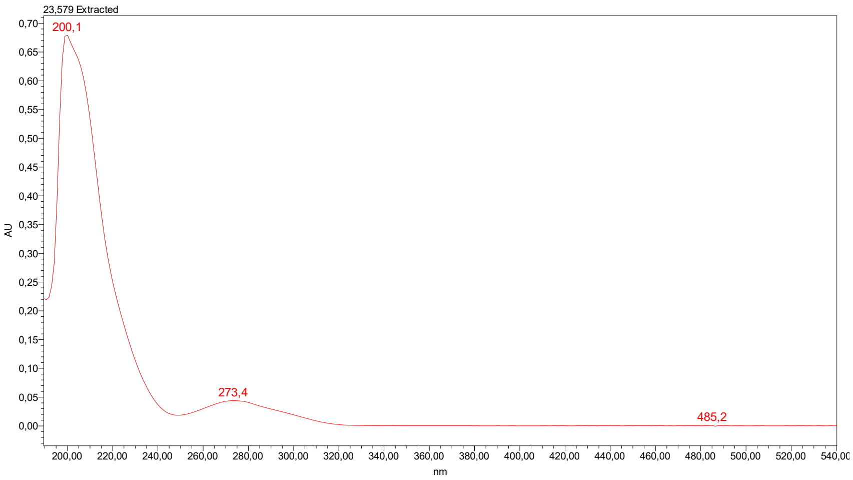

C

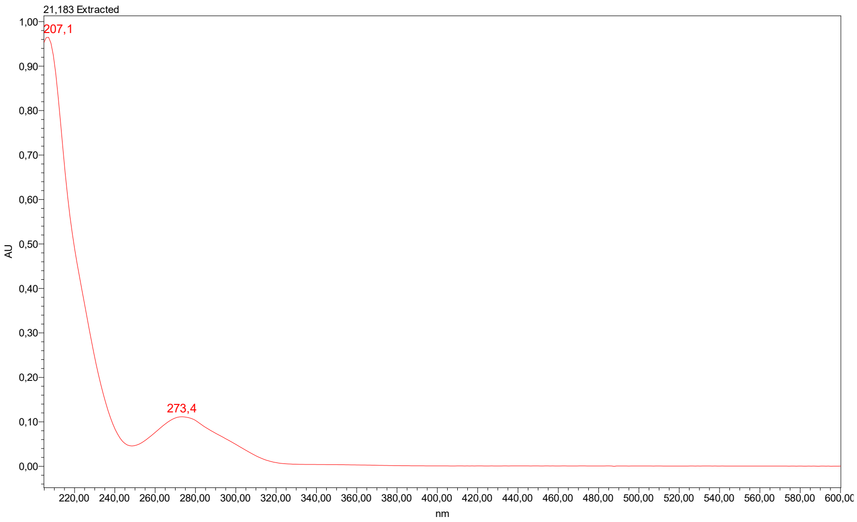

Figure S7. Chromatogram and diode array detector (DAD) UV spectra of ECG: (A) chromatogram of the ECG standard substance; (B) spectrum of the ECG standard substance; (C) representative spectra of ECG detected in the analysed samples.

A

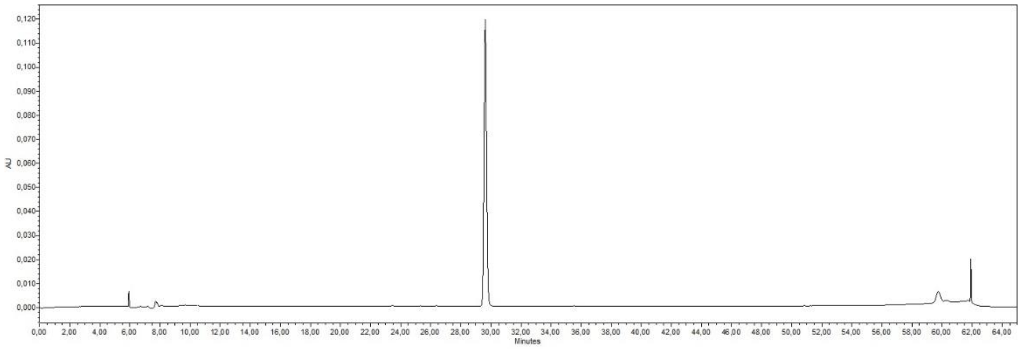

B

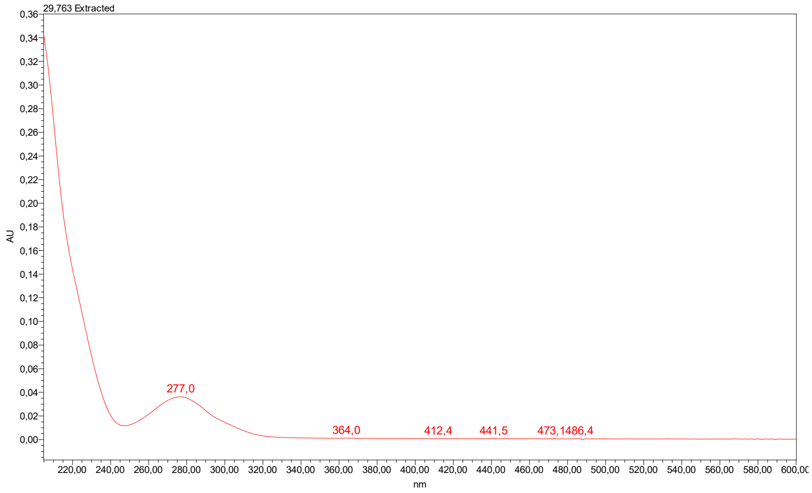

B

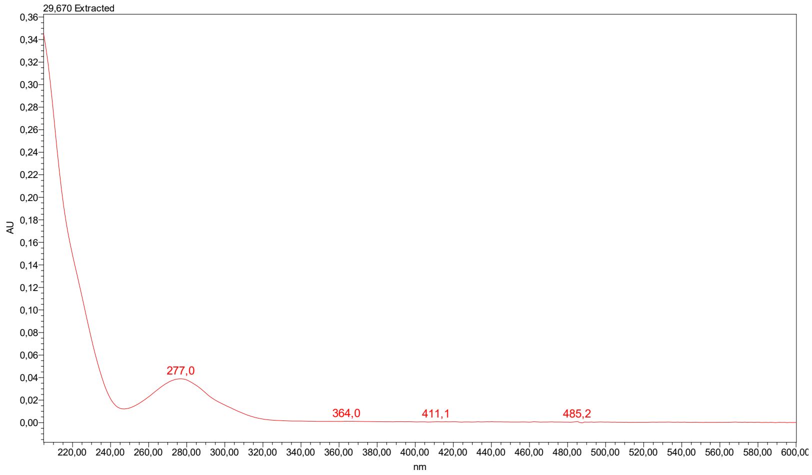

Supplement: Supplementary file 1 [file molecules-30-02789-s001.zip › molecules-3650274-supplementary.pdf]
